# Butterfly Wing Membranes’ Deformation Study

**DOI:** 10.3390/biomimetics11100717

**Published:** 2026-10-08

**Authors:** Bao Wang, Ju Qiu, Yalong Zhang, Xiangming Zheng, Kaijie Lu

**Affiliations:** 1 School of Electrical and Information Engineering, Quzhou University, Quzhou 324000, China; 2 Chengdu MCT Technology Co., Ltd., Chengdu 610011, China; 3 College of Aerospace Engineering, Nanjing University of Aeronautics and Astronautics, Nanjing 210016, China

**Keywords:** biomimetics, wing membranes, topography, dark spots, deformation

## Abstract

Flapping-wing aircraft, owing to their high aerodynamic efficiency and exceptional maneuverability, have become a focal point in bio-inspired aerospace research, with simulation serving as a critical enabler for their engineering applications. Existing studies commonly treat the wing membrane as an idealized flexible material, neglecting the significant influence of its geometric morphology on structural stiffness, and lack quantitative mechanical analysis of the membrane formation mechanism, resulting in limited simulation accuracy. This study establishes a fluid–structure coupling analysis framework by integrating prior observational data, experimental measurements, and numerical simulations. For the first time, this work shows that localized topographical thickening is capable of raising the stiffness or natural frequency of the modeled wing membrane. Findings are qualitatively consistent with select morphological features of real butterfly wings, indicate a correlation between black-spot distribution and global stiffness, and offer insights into the mechanical construction mechanism of wing membranes together with relevant optimization analysis. This stiffness-enhancing interpretation is further supported here by biochemical, morphological and geometric evidence from the published literature—melanin-coupled cuticle sclerotization, scale micro-architecture and wing corrugation—establishing the black spot as a multi-functional, stiffness-reinforcing element. This work advances the theory of morphological–mechanical coupling in flapping-wing systems, provides foundational support for high-fidelity simulation modeling, and accelerates the engineering transition of flapping-wing aircraft.

## 1. Introduction

In the early stages of human civilization, for survival, people had to observe the living habits of animals and plants and natural phenomena in the surrounding world. Therefore, the origin of bionics can be traced back to ancient times. From ancient times to the present, nature has always been humanity’s teacher. By learning from and imitating nature, humans have gradually adapted to the environment and sought survival within the natural world. Humanity has undergone a long development process from primary to advanced biomimicry, and a relatively complete disciplinary system of bionics has gradually formed in modern times.

The development of bionics can be summarized in three stages: ancient germination, modern preparation, and establishment as a formal discipline. Ancient germination: humans unconsciously imitated nature long ago—examples include Lu Ban’s wooden birds and Leonardo da Vinci’s designs for flapping-wing aircraft. Modern preparation: in the mid-20th century, the development of cybernetics, information theory and other disciplines laid a theoretical foundation for integrating biology and engineering, while military and aviation demands further promoted its progress. Establishment as a discipline: in 1958, J.E. Steele coined the term “Bionics”, and on 13 September 1960, the first U.S. symposium on bionics was held, marking the official establishment of bionics as an independent subject.

Bionics begins with reverence for nature and matures through exploration of science. Its essence is an innovative science rooted in nature. Biomimetics has already achieved numerous accomplishments, especially in its applications to aviation. There have been numerous successful bionic aircraft development cases in the past, for example, the following:

- Creature Form Bionics: The typical reference organism is the Swift (*Apus apus*), a migratory bird with an extremely streamlined external body contour that minimizes aerodynamic drag during long-duration high-speed flight. The matching aircraft is the Bionic Swift Long-endurance UAV, whose key bionic components include a curved blended wing body that perfectly simulates the swift’s smooth torso profile and a pointed, swept wingtip structure optimized by replicating the swift’s forked tail outline.
- Creature Structural Bionics: The core reference organism is the Titan Beetle (*Titanus giganteus*), whose exoskeleton features a lightweight, layered honeycomb internal structure that delivers exceptional strength with very low material density. The corresponding bionic aircraft is the Bionic Beetle Micro Unmanned Aerial Vehicle, and its main bionic structural components are a lightweight load-bearing fuselage imitating the high-toughness layered exoskeleton of the titan beetle and foldable vein-supported wing frames designed by learning from the beetle’s wing vein reinforcing structure.
- Creature Function Bionics: The representative reference organism is the Hummingbird (*Trochilidae*), which possesses the unique flight function of hovering in situ and performing ultra-low-speed maneuvering by flapping its wings at an ultra-high frequency. The targeted bionic aircraft is the Bionic Hummingbird Mini Hovering Aircraft, and its key functional bionic components include high-frequency flapping wing sets that replicate the hummingbird’s independent wing motion mechanism, as well as an omnidirectional posture adjustment module that mimics the hummingbird’s flexible tail steering function to achieve precise hovering control.

### 1.1. Flapping-Wing Bionics Research

The exploration of flapping wing flight can be traced back to ancient times. Leonardo da Vinci once sketched the design of a flapping wing aircraft, but such research developed slowly for a long time due to the limitations of aerodynamic theory and materials technology. Since the late 20th century, with the rapid progress of unsteady aerodynamics, new materials, micro-electromechanical systems and control technology, flapping wing research has entered a stage of rapid development. Flapping wing flight has obvious advantages such as high mobility, low energy consumption, high concealment and the ability to hover. It breaks through the performance limitations of traditional fixed-wing and rotary-wing aircraft and is of great significance to revealing the mechanism of biological flight and innovating aerodynamic theory. At present, flapping wing technology is widely used in micro air vehicles, battlefield reconnaissance, disaster search and rescue, environmental monitoring, aerial photography and other fields and has become an important development direction in the field of bionic aerospace.

Currently, simulation research on the flapping flight of butterflies and other insects is a highly interdisciplinary field that integrates fluid dynamics, structural dynamics, materials science, and bionic robotics technology. Its core challenges and current status are mainly reflected in the following aspects:

(1) High-Fidelity Numerical Simulation [1]: Utilizing the coupling of Computational Fluid Dynamics (CFD) and Computational Structural Dynamics (CSD), i.e., Fluid–Structure Interaction (FSI), is the mainstream research approach. Researchers strive to establish high-precision geometric models (obtained via micro-CT scanning) and simulate the wing’s flexible deformation, the generation and maintenance of the Leading Edge Vortex (LEV), and the contribution of the complex “flapping-folding-twisting” motion pattern to lift and thrust. However, high-fidelity FSI simulations incur enormous computational costs.

(2) Simplified Models and Mechanism Studies [2,3,4]: To reduce computational complexity and understand the physical essence, many studies employ simplified models, such as 2D cross-sections, rigid wings combined with prescribed pitch-flapping motions, or simplified beam/plate models to simulate wing flexibility. These studies primarily reveal the interaction between inertial and aerodynamic forces, the critical role of wing folding in reducing reverse stroke drag and enhancing vortex flow control, and the aerodynamic advantages gained from the passive storage and release of energy through flexible deformation.

(3) Experimental Validation and Data-Driven Approaches [5,6,7]: Experiments using high-speed photography, Particle Image Velocimetry (PIV), and force measurements provide crucial validation data for simulations. Simultaneously, machine learning (e.g., deep reinforcement learning) is beginning to be used to optimize flapping-wing motion trajectories and control strategies, exploring flight modes more efficient than those of biological prototypes.

(4) Bionic Robotics Drive [8,9,10]: Simulation is a key preliminary step in the design of biomimetic flapping-wing flyers (butterfly robots). The research focus lies in how to translate complex biological motion into mechanical structures realized by motors, linkages, and flexible materials, and to evaluate their aerodynamic performance and energy consumption in simulations.

However, “How can the intricate structure, materials, and motion of biological wings be transformed into a manufacturable, drivable, and controllable engineering system, and predicted and optimized using simulation tools?” The design of flapping-wing structures has evolved from “rigid” to “passively flexible,” and then to “actively controllable flexibility,” with the following characteristics across these developmental stages:

(1) Early/Simple Models [11,12]: They often employ passive flexible wings with rigid skeletons and membranes. Simulation focuses on aerodynamic performance.

(2) Current Mainstream [13]: The in-depth study of graded flexible structures (e.g., simulating veins with varying stiffness) with pre-set folds/curvature, utilizing the material’s inherent mechanical properties (e.g., anisotropy) to induce beneficial passive deformations (such as twisting, bending) under aerodynamic loads. Simulations employ FSI or simplified structural dynamics coupled with aerodynamic models.

(3) Frontier Exploration [14,15,16]: For integration of smart materials (SMA, DEA) or distributed actuators to achieve active, real-time control of wing stiffness and shape, simulations require the coupling of electro-thermal-mechanical-fluid multiphysics fields.

(4) Design and Simulation of Drive and Transmission Mechanisms [17,18]: They efficiently convert the rotary motion of motors/actuators into the wing’s complex “flapping-folding-twisting” compound motion.

(5) Design Foci [19,20]: This involves design biomimetic joints such as flexible hinges, four-bar/multi-link mechanisms, and nonlinear springs to achieve motion decoupling and energy recovery.

Recent research indicates [21] that through in vivo microscopic observation of butterfly wing scale development, scientists discovered that the formation of the ridged structure on the scale surface is controlled by mechanical buckling instability—the interaction between cell membrane growth and the constraint by actin bundles triggers the formation of ordered periodic folds. Additionally, Antónia Monteiro and co-workers [22,23,24,25,26] showed that butterfly black spots are not merely “pigment patches” but composite functional units consisting of melanin (light absorption), nano-scales (light trapping/thermal insulation/structural reinforcement), and wing veins (mechanical support). A single black spot can simultaneously perform multiple roles, including heat absorption, body temperature regulation, predator defense, and flight optimization. These functions are jointly realized by the combined structure of melanin and nano-scale architecture. The biological functions of black spots and the experimental and biomechanical evidence for their stiffness-enhancing effect are systematically reviewed in Section 1.3.

### 1.2. Current Research

In Section 1.1, recent studies have covered various aspects of flapping wings, including aerodynamic analysis, structural research, flight control, and so on. However, there has been little research on the realistic structure of flapping wing membranes and their natural formation mechanisms.

#### 1.2.1. Research Background

As the primary flight mode of natural creatures such as birds and insects, flapping-wing flight has become a research hotspot in the field of aerospace bionics owing to its advantages including high-efficiency lift generation and flexible maneuverability. It boasts broad application prospects in micro air vehicles, special detection equipment, and other fields. At present, the simulation of flapping-wing flight has become a core means to promote the engineering application of flapping-wing technology. Simulation can efficiently optimize the structural design of flapping wings, reduce experimental costs, and shorten the research and development cycle.

However, existing studies on flapping-wing simulation still have obvious limitations. On the one hand, the wing membrane is mostly regarded as a uniform, smooth, and ideal flexible structure in the simulation process, ignoring the influence of the inherent micro-topography (such as textures, folds, micropores, etc.) of biological wing membranes on membrane stiffness. This leads to deviations between simulation results and the actual flight states of creatures, making it difficult to accurately reflect the mechanical response characteristics of the wing membrane. On the other hand, the special structures of biological flapping-wing membranes (such as layered structures and fiber arrangements) are the key to their excellent mechanical properties and flight functions. Nevertheless, their natural formation mechanism (e.g., the mechanical regulation mechanism during biological growth) has not been systematically studied, and the relevant mechanical mechanisms lack quantitative verification, so they cannot provide a scientific basis for the structural design of bionic wing membranes.

Against this background, research on key issues, including the correlation between wing membrane topography and stiffness and quantitative verification of the mechanical mechanism for wing membrane structure formation, is of great theoretical significance and engineering value for improving the accuracy of flapping-wing simulation and promoting breakthroughs in bionic flapping-wing technology.

#### 1.2.2. Research Methods

This study combines biological observation, experimental testing, numerical simulation, and theoretical analysis, adopting the research logic of “biological prototype analysis—model construction—biological model—mathematical model—technical model—verification and optimization—engineering application”.

**Figure 1:**
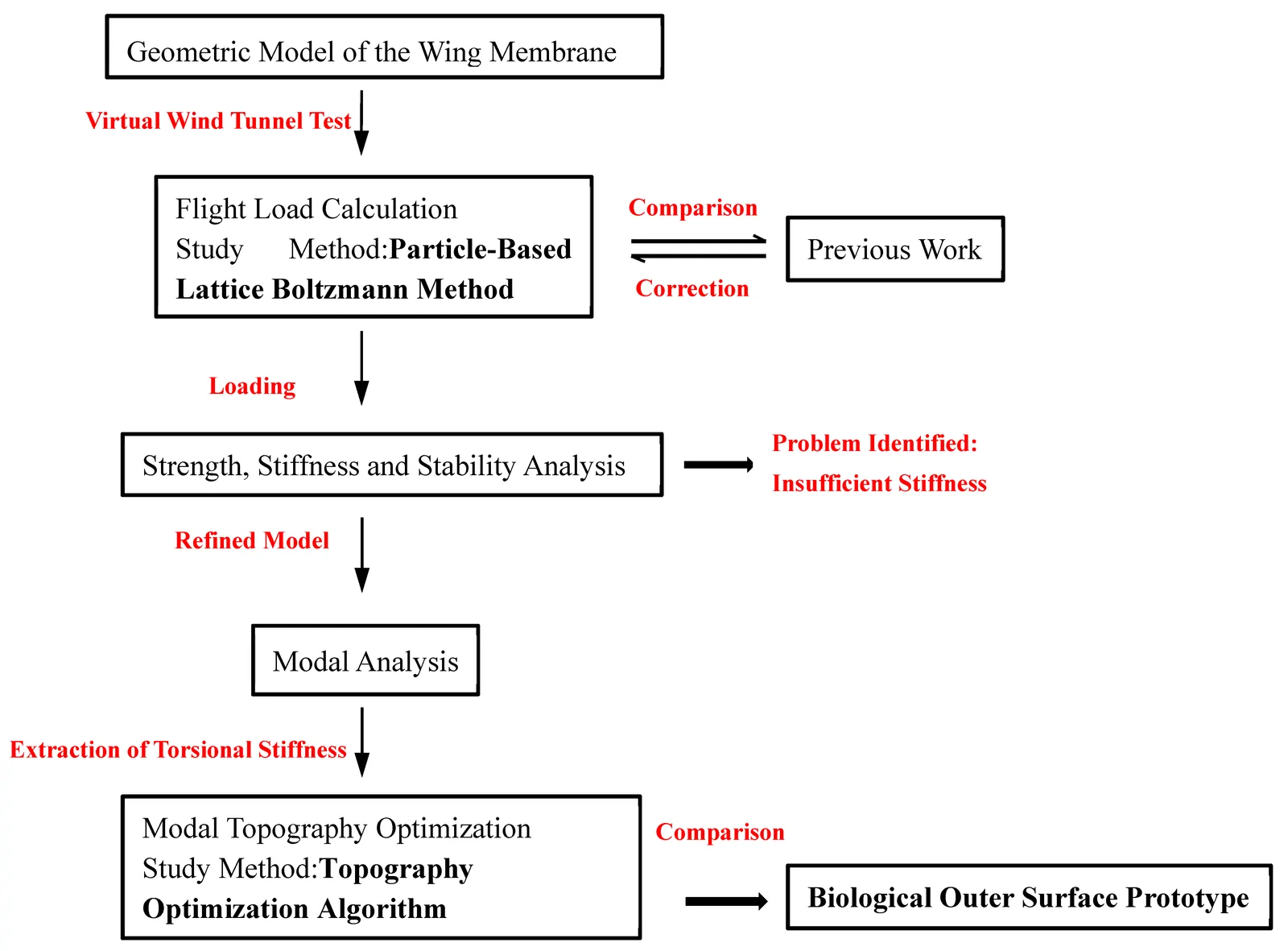
Research Flowchart.

The research in this paper is divided into three parts: flight load analysis of butterflies, structural strength and stiffness analysis of butterfly wing membranes, and topography optimization analysis of butterfly wing membranes. The specific technical route of this study is shown in Figure 1.

#### 1.2.3. Research Achievements

Our desired research achievements include the following:

- Clarify the correlation between the morphology (black spots) of flapping-wing membranes and structural stiffness, and establish a scientific and reliable morphology–stiffness correlation model for wing membranes.
- Explain the mechanical mechanism underlying the structural formation of flapping-wing membranes, and reveal the regulation law of mechanical factors on wing membrane structures during biological growth.
- Fill the research gap in the correlation analysis between morphological features and mechanical mechanisms of wing membranes in existing studies, and improve the theoretical system of flapping-wing bionics.

### 1.3. Biological Functions of Butterfly Black Spots and Evidence for Their Stiffness-Enhancing Effect

The black spots (or eyespots) on butterfly wings have long been recognized as multi-functional biological structures. Beyond their well-documented roles in pigmentation, a growing body of experimental and biomechanical evidence indicates that these dark markings also act as local structural reinforcements that increase the flexural stiffness of the wing membrane. This subsection reviews (i) the established biological functions of black spots and (ii) the biochemical, morphological and geometric mechanisms through which they enhance membrane stiffness, thereby providing the biological and experimental basis for the numerical findings presented in Section 3.

#### 1.3.1. Biological Functions of Butterfly Black Spots

Black spots, particularly eyespots, serve multiple adaptive functions in predator defense. In a controlled predation experiment with blue tits (*Parus caeruleus*), Vallin et al. [27] showed that peacock butterflies (Inachiswei io) with intact eyespots survived significantly better than those whose eyespots had been painted over: only 1 of 34 butterflies with intact eyespots was killed, whereas 13 of 20 butterflies without eyespots were killed, demonstrating that eyespots alone constitute an effective intimidation-based defense. Field experiments by Stevens et al. [28] further showed that high-contrast, concentric-ring patterns reduce predation risk even without mimicking real eyes, and the “false head” hypothesis [29] proposes that posterior-ventral wing markings deflect predatory strikes away from the vulnerable head toward the expendable hind-wing region.

Melanin-based dark spots also contribute to thermoregulation. Because melanin strongly absorbs solar radiation across a broad spectral range [30], dark wing patterns raise body temperature through radiative heating, an effect that is particularly important for flight performance and seasonal adaptation [31]. Riley [30] noted that light absorption by melanin serves photoprotection, thermoregulation, camouflage and display, while the melanin polymer itself is an important strengthening element of the insect cuticle.

In addition to defense and thermoregulation, black spots participate in sexual signaling, mimicry and species recognition, and their development, evolution and ecological significance have been extensively reviewed [22,23,26]. Importantly, these functional roles are not mutually exclusive: a single black spot is a composite functional unit in which melanin (light absorption), nano-scale cuticular architecture (light trapping, thermal insulation and structural reinforcement) and wing veins (mechanical support) act together [22,23,24,25,26].

#### 1.3.2. Evidence That Black Spots Enhance Wing Membrane Stiffness

Biochemically, melanin synthesis and cuticle sclerotization (hardening) share the same tyrosine-derived metabolic pathway [30,32]. During sclerotization, N-acyl dopamine derivatives such as NBAD (N-β-alanyldopamine) and NADA (N-acetyldopamine) are oxidized by the phenoloxidase laccase 2 into quinones and quinone methides that cross-link cuticular proteins, rendering the cuticle insoluble, harder and darker [32,33]. In the red flour beetle Tribolium castaneum, RNA-interference silencing of laccase 2 abolished cuticle tanning and produced soft, deformed individuals [32], while dynamic mechanical analysis of “black” cuticle mutants revealed reduced cross-linking of cuticular components and altered mechanical properties [33]. These results establish that melanin pigmentation and cuticular hardening are mechanistically coupled: dark (melanin-rich) regions are generally more thoroughly sclerotized and therefore mechanically stiffer than unpigmented regions. As Riley [30] summarized, melanin is “an important strengthening element of … insect cuticle.”

Morphologically, the melanin pathway also regulates the fine architecture of wing scales. Using CRISPR/Cas9 knockout, Matsuoka and Monteiro [34] showed that mutations in melanin-pathway genes (e.g., yellow and DDC) alter both pigmentation and the fine structure of wing scales—for example, producing extra horizontal laminae or taller sheet-like vertical laminae—demonstrating that scale morphology and coloration are genetically linked. Consequently, the scales covering black spots typically exhibit denser, thicker or more elaborate cuticular microstructures than the surrounding light-colored scales, providing a morphological basis for local stiffness enhancement. Microscopic observations further indicate that the wing membrane at eyespot foci is physically distorted and structurally distinct from the surrounding regions [35], consistent with a localized mechanical reinforcement.

Geometrically, the stiffness-enhancing role of localized thickening and corrugation is well established in insect wing mechanics. Numerical analysis of corrugated airfoils demonstrated that corrugation substantially increases resistance to bending, and that a smooth structure would have to be roughly three times thicker to provide the same bending resistance [36]. The V-shaped corrugations of insect wings stiffen the wing against spanwise bending while allowing torsion [36,37], and the arrangement of wing veins increases the fracture toughness of the locust hind wing by about 50% [38]. Wootton [39] emphasized that wing relief—corrugation, camber and membrane tension—is fundamental to supporting the wing during flight. Finite-element simulations of the ridge-and-cross-rib microstructure of butterfly wing scales further showed that the curved, bio-realistic geometry distributes stress more evenly and yields better mechanical performance than simplified rectangular geometry [40]. These independent lines of evidence converge on the same principle: local geometric relief and thickening constitute an efficient, low-mass strategy to raise the bending stiffness and natural frequency of a thin membrane—precisely the behavior reproduced by the topography-optimized profile of the present study (Section 3.2.4).

Taken together, the black spots of butterfly wings should be regarded not merely as pigment patches but as multi-functional, stiffness-reinforcing elements in which pigmentation, scale morphology, membrane thickening and venation act synergistically. This functional integration provides a biologically grounded explanation for the numerical result that local thickening at black-spot-like locations increases membrane stiffness and natural frequency, and offers a design principle for the morphology–stiffness coupling of biomimetic flapping-wing membranes.

## 2. Analysis Methods

The formation of butterfly wing membranes involves multiple disciplines. Focusing on the flight load, structural strength, stiffness and stability of wing membranes, this study investigates the enhancement of wing membrane stiffness through morphological optimization and confirms that such stiffness improvement is closely related to the protruding black spots on the membrane surface. This study investigates the natural formation of butterfly wing membranes through aerodynamic load analysis of the wing surface, structural static, buckling analysis and dynamic analysis, and by employing topography optimization methods.

### 2.1. Particle-Based Lattice Boltzmann Method

This method does not use traditional mesh generation. Instead, it discretizes the fluid domain into a series of Lagrangian particles. These particles move and collide according to specific rules on Eulerian grid points. Its core is solving the mesoscopic Lattice Boltzmann equation.

#### 2.1.1. Core Evolution Equation

The discretized equation governing fluid evolution is

*f_i_*(*x* + *e_i_*∆*t*, *t* + ∆*t*) − *f_i_*(*x*, *t*) = Ω*_i_*(*x*, *t*) (1)

where *f_i_* is the particle distribution function, the core variable in LBM, representing the density of particles moving with a discrete velocity **e***_i_* at position x and time t; **e***_i_* is the discrete velocity direction; and Ω*_i_* is the collision operator, representing the change in the distribution function due to particle collisions.

#### 2.1.2. Key Improvement: The Central-Moments Collision Operator

A significant technical feature of XFlow is its innovative implementation of the collision operator. Unlike traditional Multiple Relaxation Time (MRT) models, XFlow’s scattering operator is implemented in central-moments space:

Ω*_i_* = *M*^−1^*S*(*Mf_i_* − *m_eq_*) (2)

where M is the transformation matrix used to map the distribution function *f_i_* to central-moments space m; S is the relaxation matrix, controlling the rate at which each moment relaxes towards its equilibrium state; and *m_eq_* is the equilibrium central moments. Technical advantages: performing collisions in central-moments space significantly enhances the algorithm’s Galilean invariance, numerical stability, and accuracy, which is crucial for accurately simulating the high-frequency, unsteady flows generated by rotating components.

#### 2.1.3. Calculation of Macroscopic Quantities

The macroscopic physical quantities of the fluid, such as density ρ and velocity u, are obtained by summing the distribution functions:

(3) $$\rho =\sum_{i}f_{i}\;,\;\;\;\;\rho u=\sum_{i}f_{i}e_{i}$$

Pressure *p* is directly linked to density via the equation of state: $p=c_{s}^{2}\rho$, where *c_s_* is the lattice speed of sound.

### 2.2. Buckling Analysis

For eigenvalue buckling analysis, a classical numerical method is used to predict the critical buckling load and corresponding buckling mode shape of thin-walled structures, beams, plates, shells and bionic wing membranes under compressive load. It is based on small deformation, linear elasticity and geometric linear assumptions, ignoring the influence of large deflection, material nonlinearity and initial imperfections.

#### 2.2.1. Governing Equilibrium Equation

The static equilibrium equation of the structural system under external load is defined as follows:

([*K_E_*] + *λ*[*K_G_*])*ϕ_i_* = 0 (4)

Solve the characteristic equation

*det*([*K_E_*] + *λ*[*K_G_*]) = 0 (5)

where *K_E_* is an elastic stiffness matrix of the structure; *K_G_* is a geometric stiffness matrix (initial stress stiffness matrix); *λ_i_* is a buckling load factor (eigenvalue) of the i-th order; *ϕ_i_* is i-th order buckling mode vector (eigenvector).

Solving the equation yields a series of ordered eigenvalues and mode shapes:

*λ*_1_ < *λ*_2_ < *λ*_3_ <...< *λ_n_*; *ϕ*_1_, *ϕ*_2_, *ϕ*_3_,...,*ϕ_n_*.

#### 2.2.2. Critical Load for Higher-Order Buckling

*P_cr_*_,*i*_ = *λ_i_*∙*P_ref_* (6)

where *P_ref_* is applied reference load and *P_cr_*_,*i*_ is critical load of the i-th higher-order buckling mode.

The first-order (i = 1) is the fundamental buckling mode with the minimum critical load; modes with i ≥ 2 are defined as higher-order buckling. If *λ* < 0, the calculated critical load *P_cr_* becomes tension.

For conventional structural stability problems dominated by compression, negative eigenvalues possess no practical engineering significance and are usually discarded directly. Only the minimum positive eigenvalue is adopted to compute the critical compressive buckling load for linear problems. Higher-order buckling corresponds to the subsequent eigenvalue solutions beyond the first-order fundamental mode. Each higher-order eigenvalue *λ_i_* represents an elevated critical load required to trigger a more complex structural buckling deformation. Higher-order mode shapes exhibit sophisticated spatial deformation characteristics, such as multi-wave buckling waveforms, local wrinkling, antisymmetric deflection, and combined bending-torsion deformation, which are more intricate than the global single-wave deformation of the first-order mode. Physically, higher-order buckling modes cannot be spontaneously activated under normal monotonic compression. They can only be excited under extreme load levels, combined loading, initial geometric imperfections, boundary constraint variation, or dynamic excitation. In structural engineering and bionic thin-wing membrane analysis, higher-order buckling modes are widely adopted to establish initial imperfection fields, conduct post-buckling simulation, and evaluate structural ultimate bearing capacity and multi-mode stability failure.

### 2.3. Topography Optimization Algorithm Method

Regulating the vibration frequency of a structure by optimizing its own shape (topology) [41,42] is a cutting-edge field deeply intersecting mechanics, mathematics, and engineering design. The “topography optimization” discussed here actually refers to a branch of Topology Optimization. Its core idea is to find the optimal thin-walled structural shape within a given design space, so as to maximize specific natural frequencies (e.g., the fundamental frequency) of the structure or to meet design requirements for frequency separation.

#### 2.3.1. Optimization Principle

The natural frequency ω of a thin-walled structure (such as an aircraft wing panel, car door, butterfly wing membrane, etc.) is determined by the Rayleigh quotient:

(7) $$\omega^{2}=\frac{\mathrm{Modal}\;\mathrm{Stiffness}}{\mathrm{Modal}\;\mathrm{Mass}}=\frac{u^{T}\mathrm{Ku}}{u^{T}\mathrm{Mu}}$$

where u is the mode shape vector corresponding to the mode, K is the structural stiffness matrix, and M is the structural mass matrix. The core principle of the optimization is, under the premise of minimizing mass increase, to maximize the generalized stiffness of the structure for that specific mode by redistributing material (e.g., adding stiffeners in areas with large vibrational deformation, thinning, or creating holes in areas with small deformation). This is equivalent to changing the “topography” of the structure—the distribution of peaks and valleys determines the force transmission path and the ability to resist deformation.

#### 2.3.2. Mathematical Formulation

The precise mathematical formulation of this problem within the continuum framework is provided below. This is a standard formulation based on Free-Form Optimization:

(1) Problem Definition: Consider a thin-walled structure with its mid-surface A and constant thickness h. The goal is to find the optimal mid-surface shape (i.e., the shape variation) *Ω_s_* that maximizes the k-th order natural frequency *ω_k_* of the structure.

(2) Complete Optimization Model:

(8) $$\begin{array}{c} \underset{\Omega }{\max}\;\;\lambda_{k}=\omega_{k}^{2}\left(\mathrm{Maximize}\;\mathrm{the}\;k-\mathrm{th}\;\mathrm{eigenvalue}\right)\\ \;\;\;\;s.t.\;\;\int_{\Omega }\rho h\;\mathrm{dA}\;\;\leq \;M^{*}\;\;\left(\mathrm{Mass}\;\mathrm{Constraint}\right)\\ \mathrm{and}\;\mathrm{the}\;\mathrm{physical}\;\mathrm{equation}: \end{array}\;\underset{\mathrm{Virtual}\mathrm{Work}(\mathrm{Stiffness}\mathrm{Term})}{\underbrace{\int_{A}\left(\delta \varepsilon_{m}^{T}C_{m}\varepsilon_{m}+\delta \varepsilon_{b}^{T}C_{b}\varepsilon_{b}\right)\mathrm{dA}}}-\underset{\mathrm{Inertial}\mathrm{Term}}{\underbrace{\omega^{2}\int_{A}\rho h\delta u\cdot \mathrm{udA}=0,}}\;\;\forall \delta u\in U$$

where *λ_k_* is the square of the k-th natural frequency (maximizing *λ_k_* is equivalent to maximizing the dynamic stiffness for that mode); the membrane strain energy and bending strain energy terms are, respectively, the strain energies caused by in-plane stretching and out-of-plane bending; the inertial term represents the influence of the structure’s mass distribution on vibration; the mass constraint limits the total mass to a given value M*; AND U is the space of admissible displacement fields satisfying the boundary conditions.

#### 2.3.3. Core Mechanism: Shape Gradient and Variation

The optimization problem above cannot be solved directly; it must be transformed into an iterative process. The key lies in solving for the shape gradient function G. This function indicates, for the current design, how to move the surface to decrease the objective function most effectively. For eigenvalue maximization problems, the general form of the shape gradient is:

G = (Local Energy Density) − (Curvature-related Term) × (Objective Value)(9)

Specifically, in free-form optimization, the design velocity field V (i.e., the infinitesimal displacement of the surface along its outward normal) is determined by the following equation:

(10) $$a\left(V,\;\;\bar{V}\right)=-\left(G,\;\;\;\;\bar{V}\right),\;\;\;\;\;\;\;\forall \bar{V}\in C_{\theta }$$

where a(·,·) is an artificially defined smoothing metric (e.g., the bilinear form of linear elasticity for thin shells), used to ensure the updated surface remains smooth; G is the shape gradient function (sensitivity information), derived from adjoint variable methods and material derivatives; and $\bar{v}$ is the space of admissible variations.

Optimization iteration process: (1) solve the current eigenvalue problem to obtain the frequency *ω_k_* and mode shape *u_k_* for the current shape; (2) compute the shape gradient G based on the distribution of strain energy and kinetic energy from the current mode shape; (3) solve for the velocity field V by converting the shape gradient G into a smooth surface update velocity field using the smoothing metric equation; (4) update the shape: *Ω_new_* = *Ω_old_* + V. Repeat steps 1–4 until convergence.

#### 2.3.4. Specialized Treatment for Thin-Walled Structures

The specific characteristic of thin-walled structures lies in their thickness h being much smaller than other dimensions. Two main strategies are often used in optimization:

(1) Stiffener Layout Optimization: distribute stiffeners on a base plate to alter local bending stiffness. Here, the design variables are the presence/absence and thickness of the stiffeners. An example of a material interpolation model (e.g., polynomial interpolation) is defined below:

(11) $$E_{e}\left(T_{e}\right)=\left[5{\left(\frac{T_{e}}{T_{m}}\right)}^{5}+\frac{T_{e}}{T_{m}}\right]E_{0}\;\;\;\;\;\;\;\left(0\leq T_{e}\leq T_{m}\right)$$

where *T_e_* is the stiffener thickness per element, and *T_m_* is the maximum thickness.

(2) Free-Form Shape Optimization: directly alters the surface shape of the mid-surface, where the design variables are the coordinates of all nodes. The present study investigates the natural formation of the butterfly wing membrane shape using this approach.

Essentially, this research tackles a shape optimization problem aimed at maximizing the k-th order eigenvalue under mass and governing equation constraints. Its core lies in a shape gradient-driven iterative process that automatically aggregates material towards regions most effective in enhancing modal stiffness for that specific order, while ensuring a smooth and continuous surface. In recent years, advances in bio-inspired algorithms, free-form shape optimization, and methods for handling multiple eigenvalues like the cluster mean value method have demonstrated the powerful engineering value of this tool in fields such as aerospace and automotive industries.

## 3. Case Study for Butterfly Wing Membrane Formation

### 3.1. Analysis of Butterfly Flight Loads

**Figure 2:**
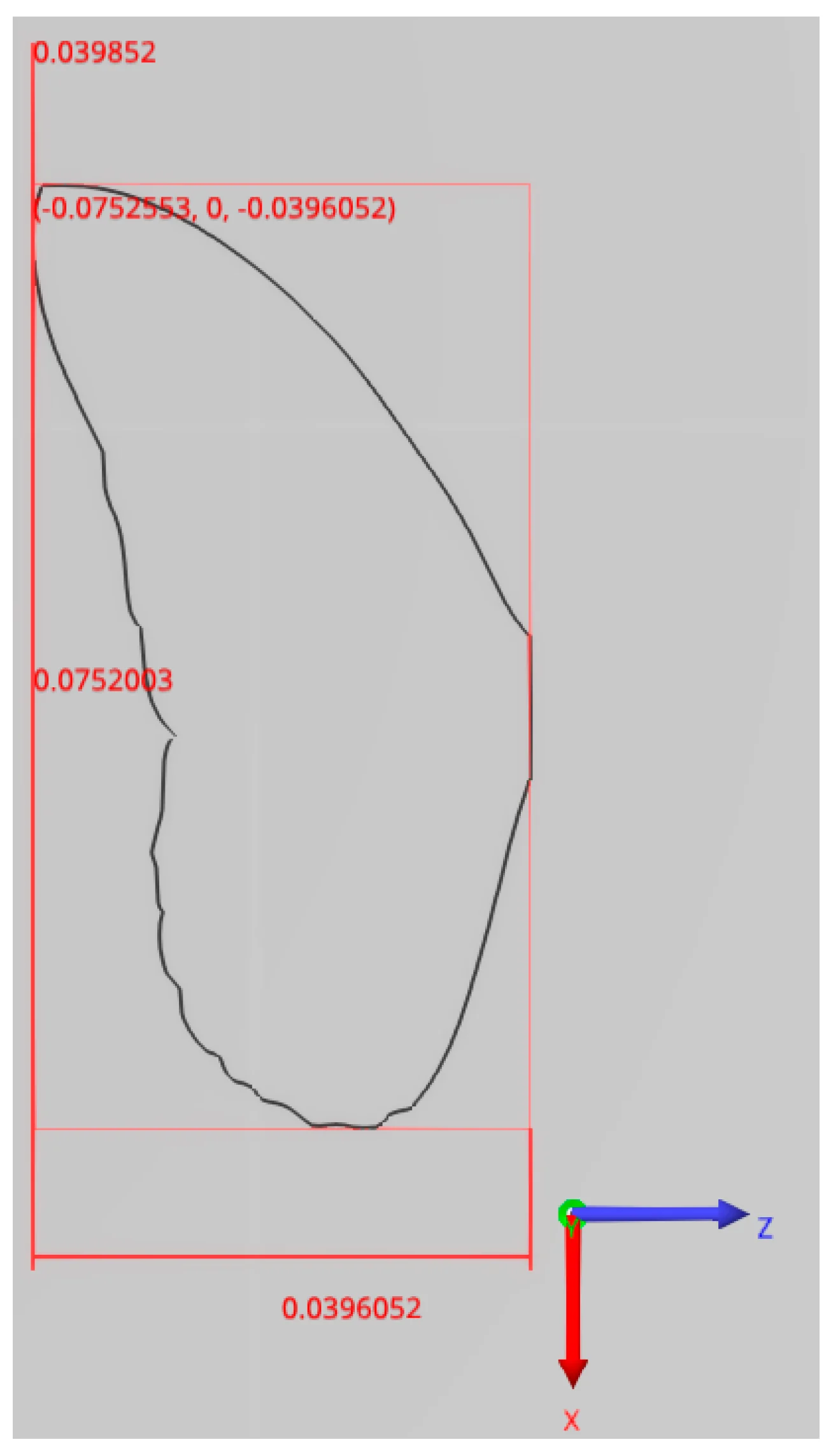
Dimensional Drawing of Butterfly Geometry (unit: m).

The dimensional specifications of the butterfly are shown in Figure 2.

**Figure 3:**
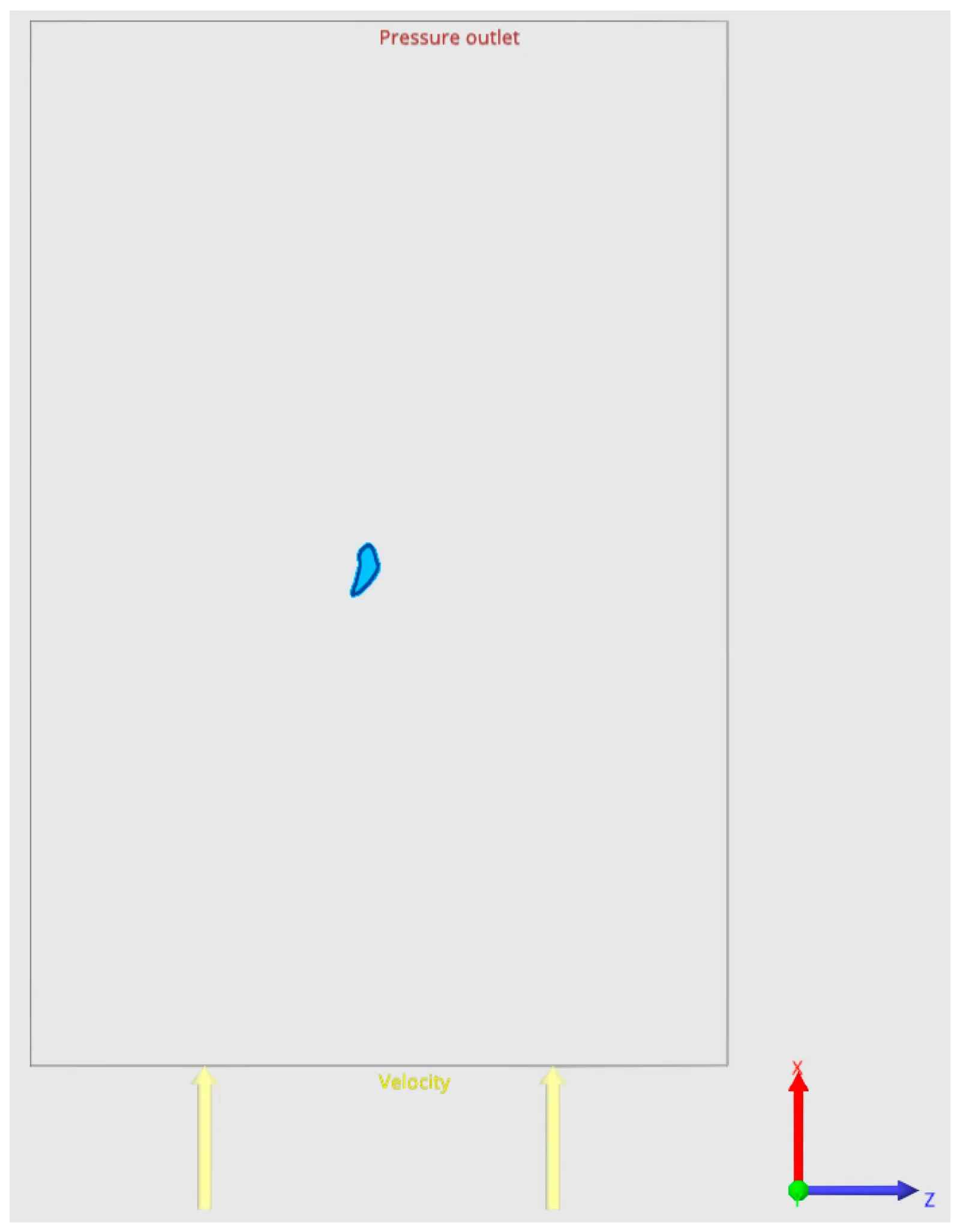
Simulation Wind Tunnel for Butterfly Flight Loads.

The configuration of the butterfly in the simulation wind tunnel is shown in Figure 3.

The inlet of the flow field is prescribed with a velocity boundary condition, and the outlet with a pressure boundary condition. The inflow air velocity is defined as 1.88 m/s. It is assumed that the biological butterfly wing surface undergoes a periodic flapping motion, essentially revolving around the fuselage plane with an initial angle of 90° and a flapping amplitude ranging from −60° to 90°. The positional change approximates a trigonometric function, meaning the relationship between the wing surface angle and the fuselage horizontal plane can be expressed by the following equation:

(12) $$\left\{\begin{array}{ll} \varphi \left(t\right)= & \varphi_{0}+\frac{A}{2}\cos\left(2\pi \mathrm{ft}\right)\\ & \varphi_{0}=15^{0} \end{array}\right.$$

**Figure 4:**
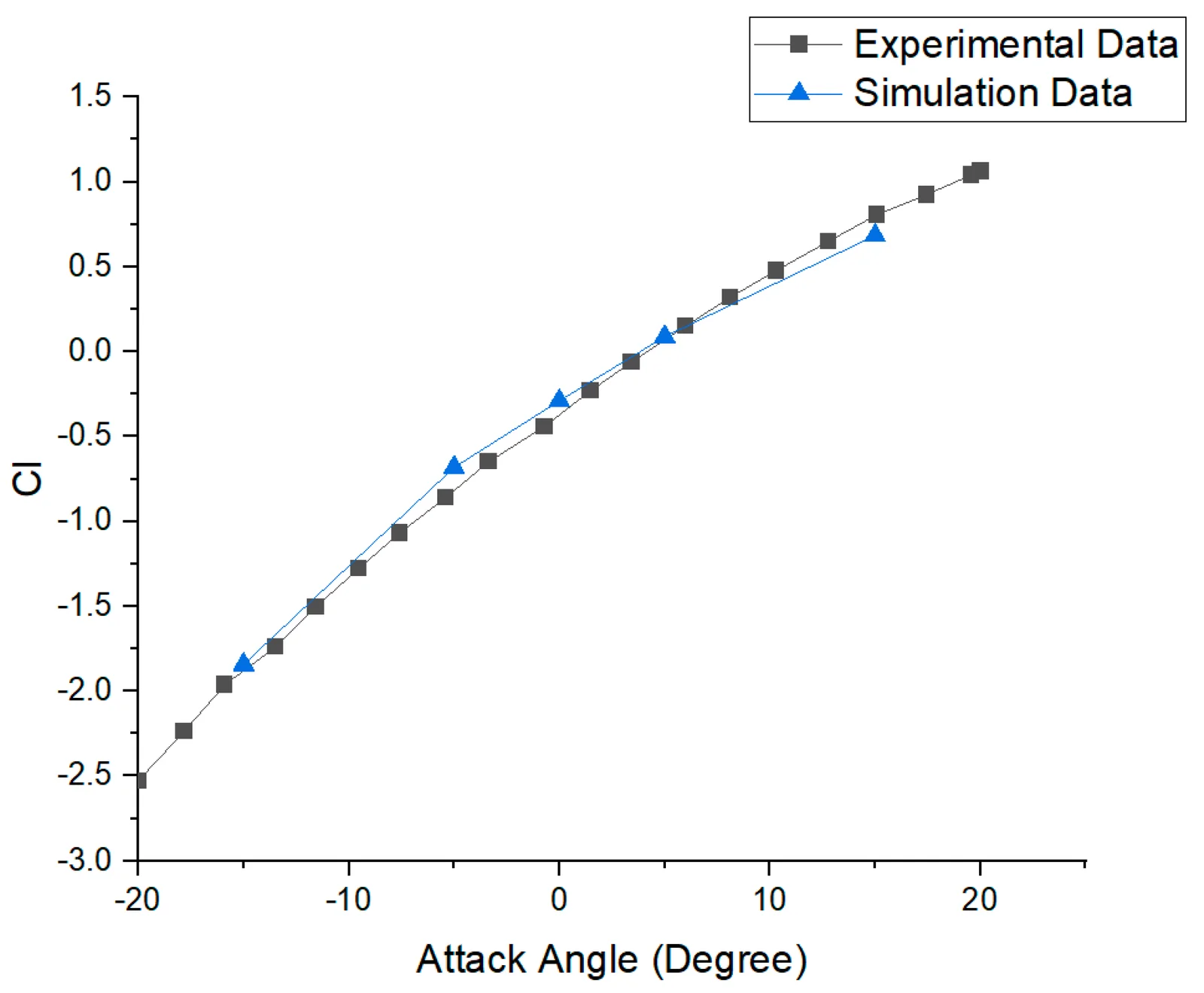
Comparison Chart of Lift Coefficients.

**Figure 5:**
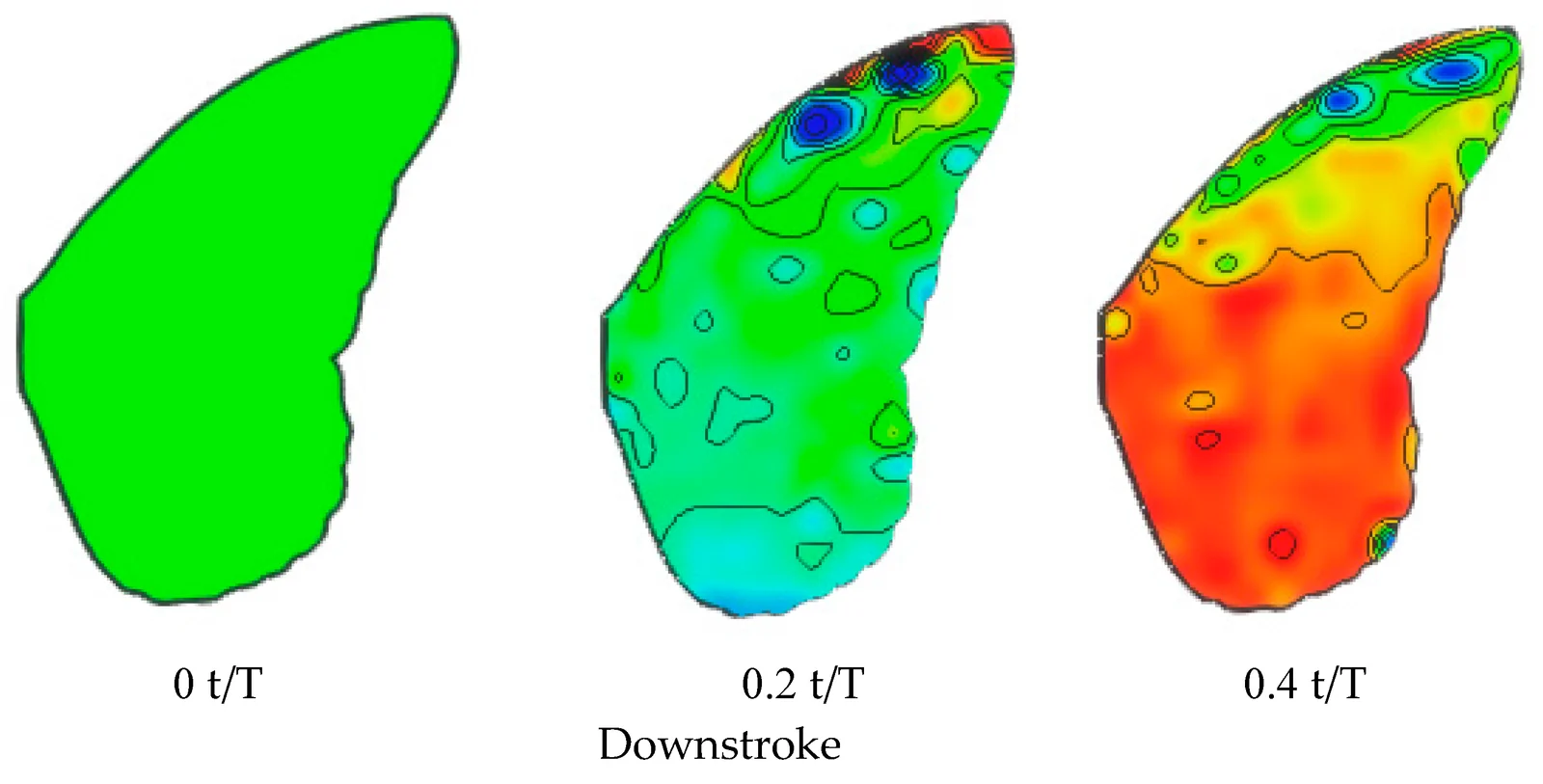

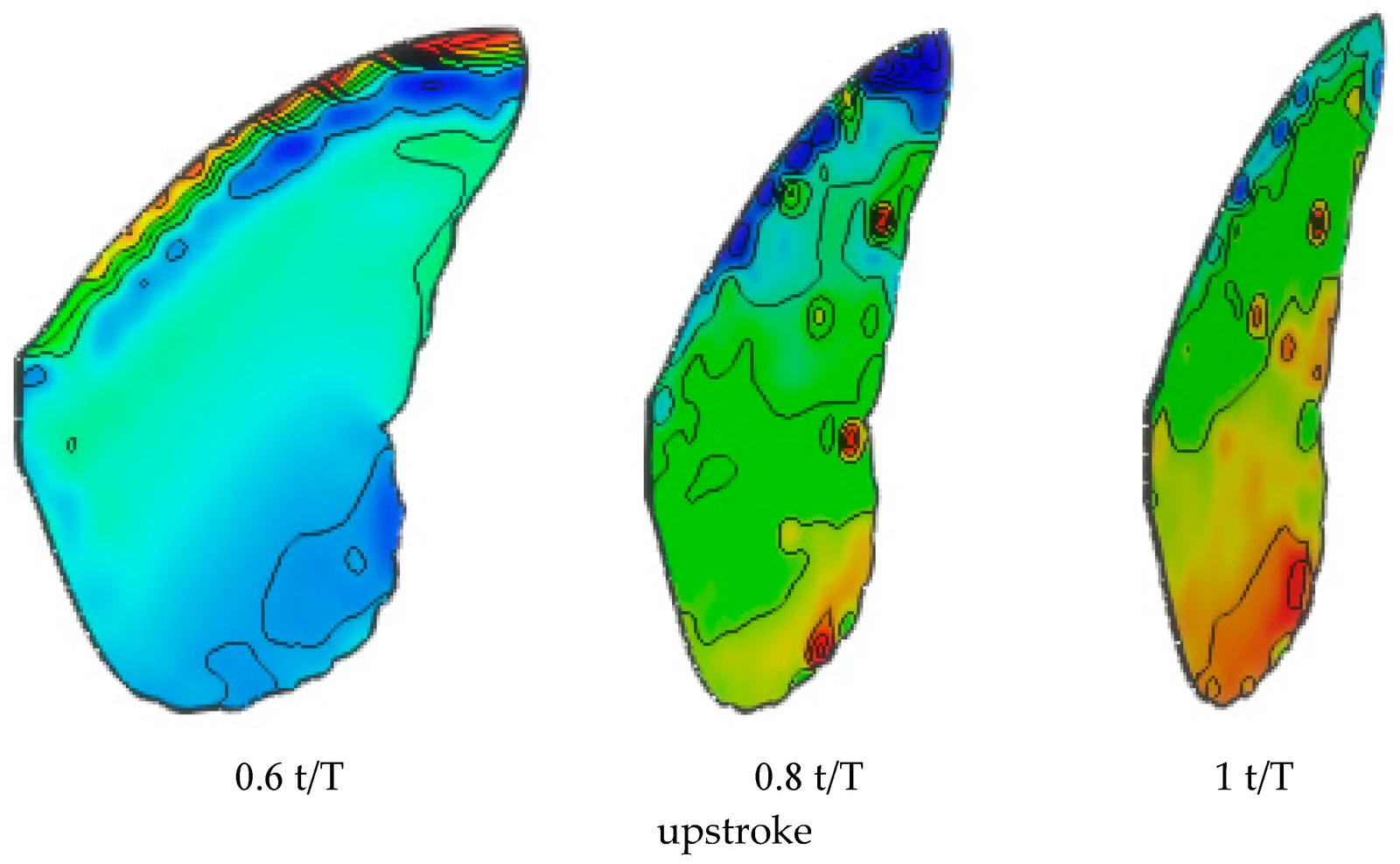
Pressure Distribution Diagrams of the Simulated Butterfly Wing Membrane.

**Figure 6:**
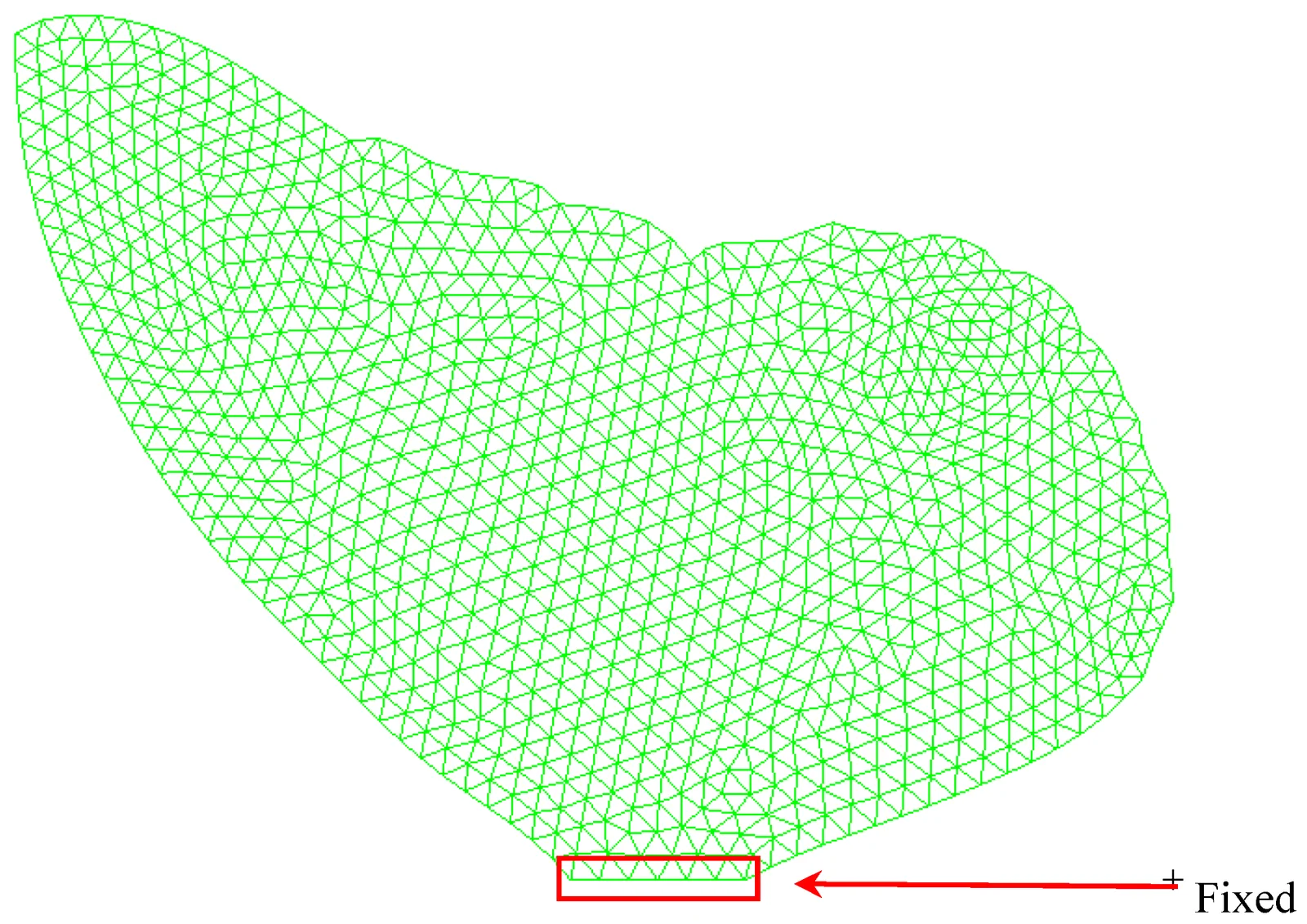
Finite Element Model of the Butterfly Wing Membrane Structure.

In the equation, *φ*(*t*) is the wing flapping angle, *φ*_0_ is the initial angle, *A* is the flapping amplitude (A = 150°), *f* is the flapping frequency, and *t* is time. The angle of attack of the simulated butterfly is modeled using a sinusoidal function, with an amplitude ranging from −5° to 5° (please see Figure 5 and Table 3 of Ref. [43]). For the simulation of the butterfly’s up-and-down flapping motion, refer to Reference [44]. A comparison of the lift coefficient with the butterfly shape from Figure 6 in Reference [43] is shown in Figure 4 below.

The experimental peak lift coefficient is 0.81, while the simulation analysis yields 0.688 (The computation at a 15° angle of attack was added as supplementary work), resulting in an error of −15%.

The pressure distribution diagrams of the simulated butterfly wing membrane at various typical moments during its rotation in the flow field are shown in Figure 5.

### 3.2. Analysis of Butterfly Structural Strength and Modal Optimization

The material mechanical parameters of the butterfly structure are listed in Table 1. Since this round is a preliminary exploration of the structural formation of butterfly wing membranes, the large-size parameters listed in the table below are adopted to facilitate the multidisciplinary optimization research.

#### 3.2.1. Structural Finite Element Model

The finite element model of the butterfly wing membrane structure is shown in Figure 6. During static analysis, the part of the wing membrane connected to the butterfly’s body is constrained with fixed support constraints.

**Figure 7:**
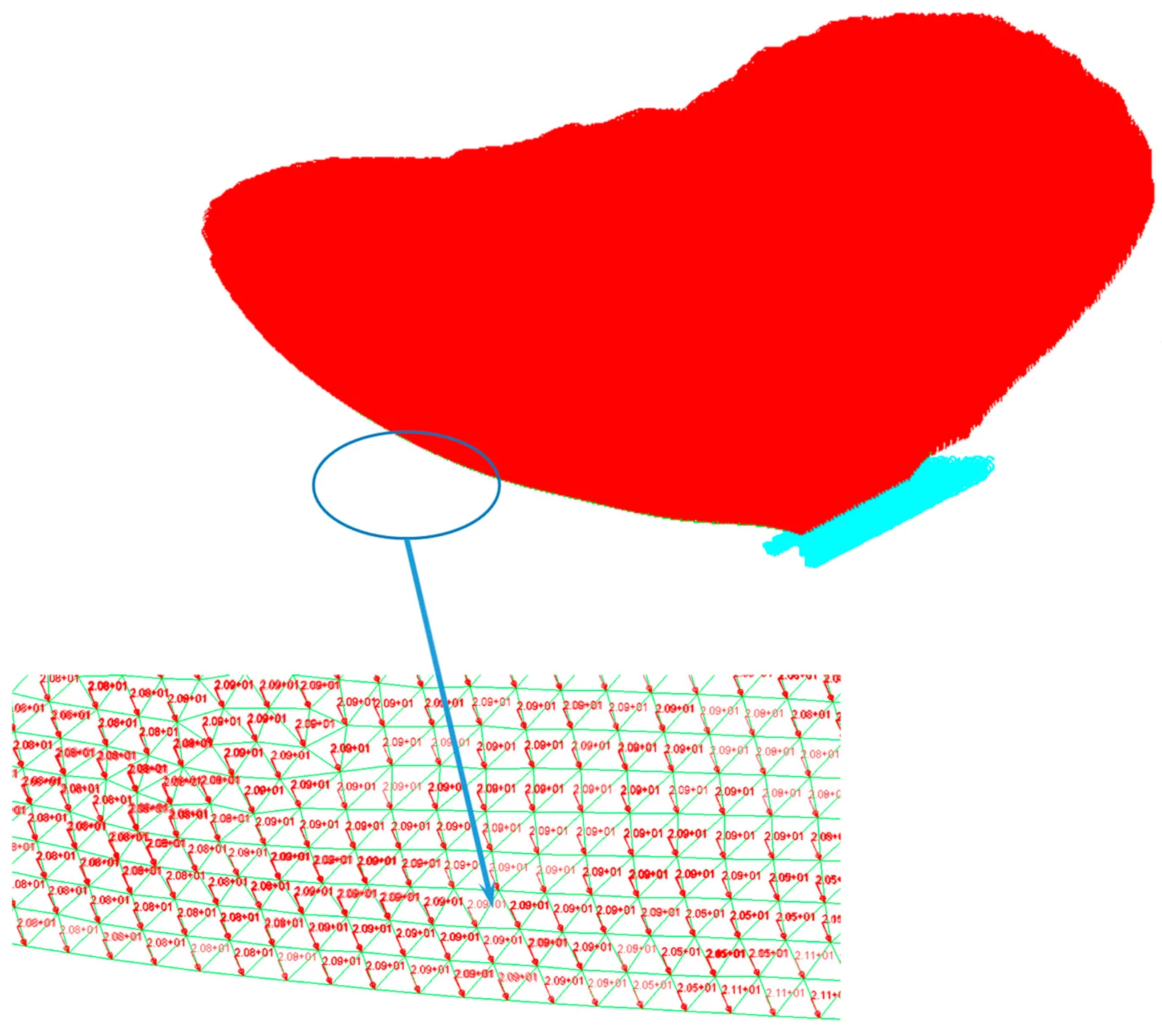
Loading Finite Element Model of the Butterfly Wing Membrane Structure (Pa).

The finite element model contains 1041 nodes and 1962 elements. After mesh adjustment, the element size is uniform, with nearly all elements approximating equilateral triangles to ensure the convergence and correctness of all simulation analyses. Further discussion on the convergence of the mesh calculation simulation can be found in the stress contour plot section of Section 3.2.2 below. A MATLAB 7.0 script was used to map the static pressure described in Section 3.1 onto the structural nodes, as illustrated in Figure 7.

#### 3.2.2. Structural Strength and Stiffness Analysis

The highest pressure distribution extracted from Section 3.2.1 was converted via program to form the load case for the static analysis. After submitting the calculation, the support reaction forces are listed in Table 2.

As the extracted pressure is perpendicular to the butterfly wing membrane and the membrane has a rotational angle with respect to the original coordinate system, the reaction force contains Y and Z components.

**Figure 8:**
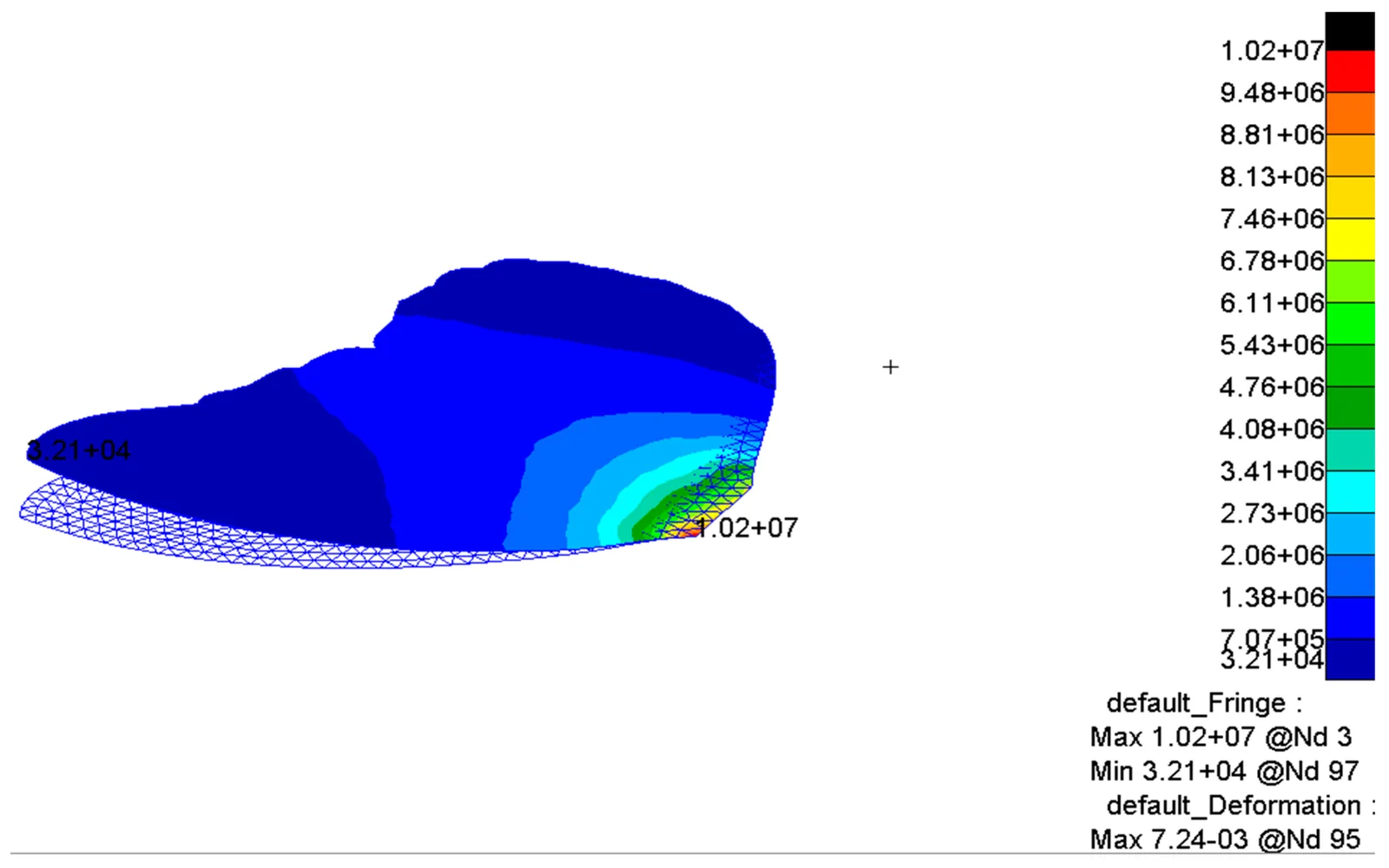
Simulation Deformation and Stress Diagram of Butterfly Wing Membrane (Pa & m).

**Figure 9:**
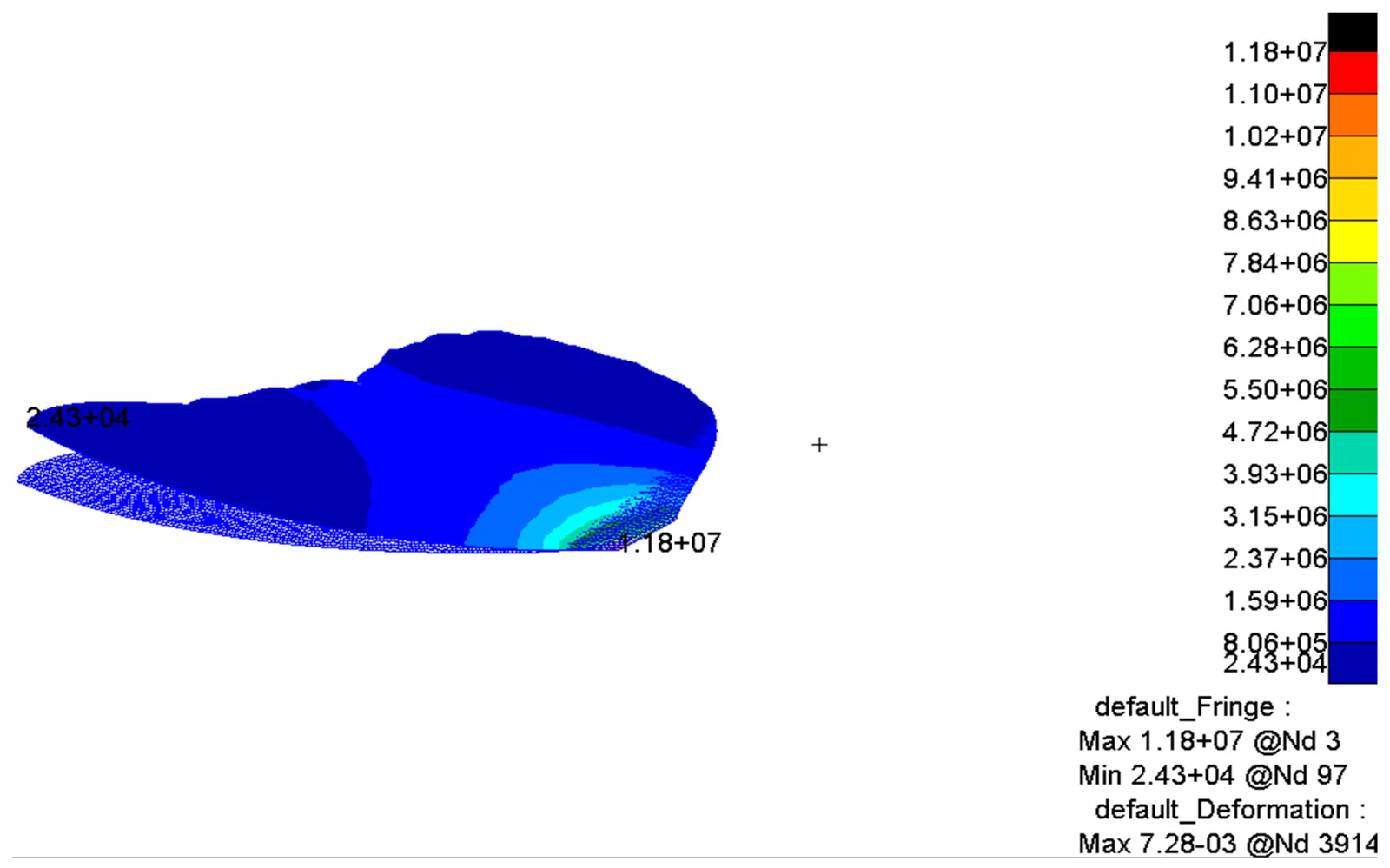
Simulation Deformation and Stress Diagram of Butterfly Wing Membrane (Pa & m).

**Figure 10:**
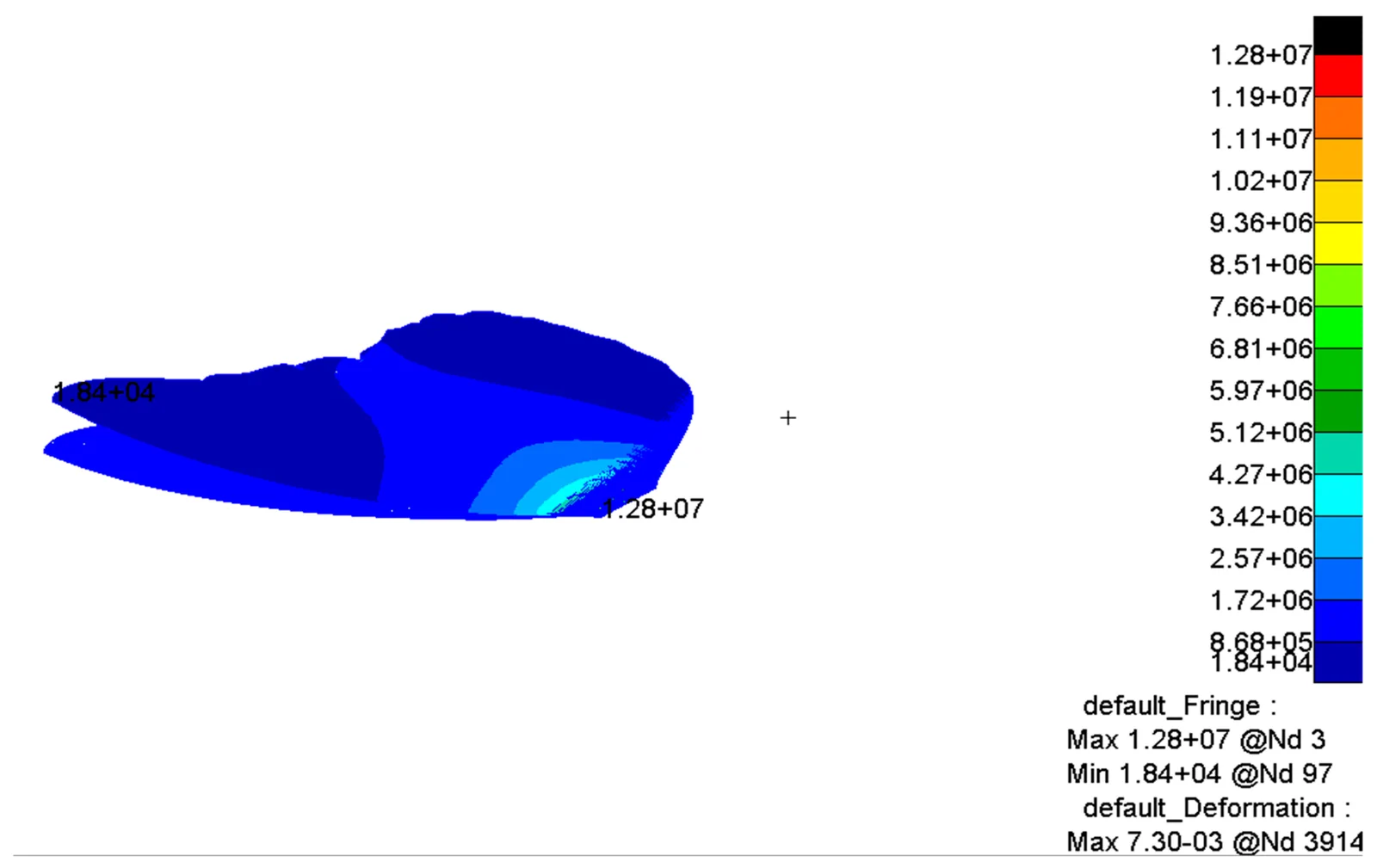
Simulation Deformation and Stress Diagram of Butterfly Wing Membrane (Pa & m).

To investigate the mesh convergence of the finite element model, mesh refinement was performed at different levels. Under identical loads and boundary conditions, computational analysis yielded the contour plots and deformation states presented in Figure 8 (coarse mesh), Figure 9 (medium mesh) and Figure 10 (fine mesh).

Based on the analysis results, Table 3 was compiled.

As shown in the above table, the displacement simulation error between the medium and fine meshes is around 0.2%. Hence, the fine mesh solution is regarded as converged and used for subsequent analyses.

As can be seen from the figure above, the deformation of the simulated wing membrane structure is coordinated and consistent. The deformation at the wing tip is relatively large, reaching 7.3 mm, which is close to half of the simulated maximum butterfly chordwise length. This indicates that the stiffness of the wing membrane plane itself is insufficient. Figure 10 shows the stress contour plot, where the maximum stress at the root is merely 12.8 MPa.

#### 3.2.3. Structural Stability Analysis

**Figure 11:**
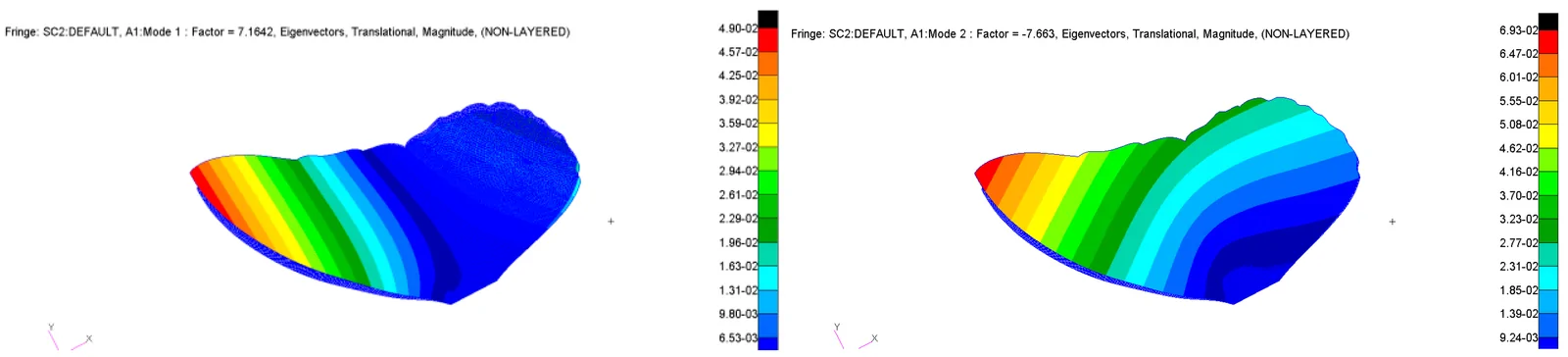

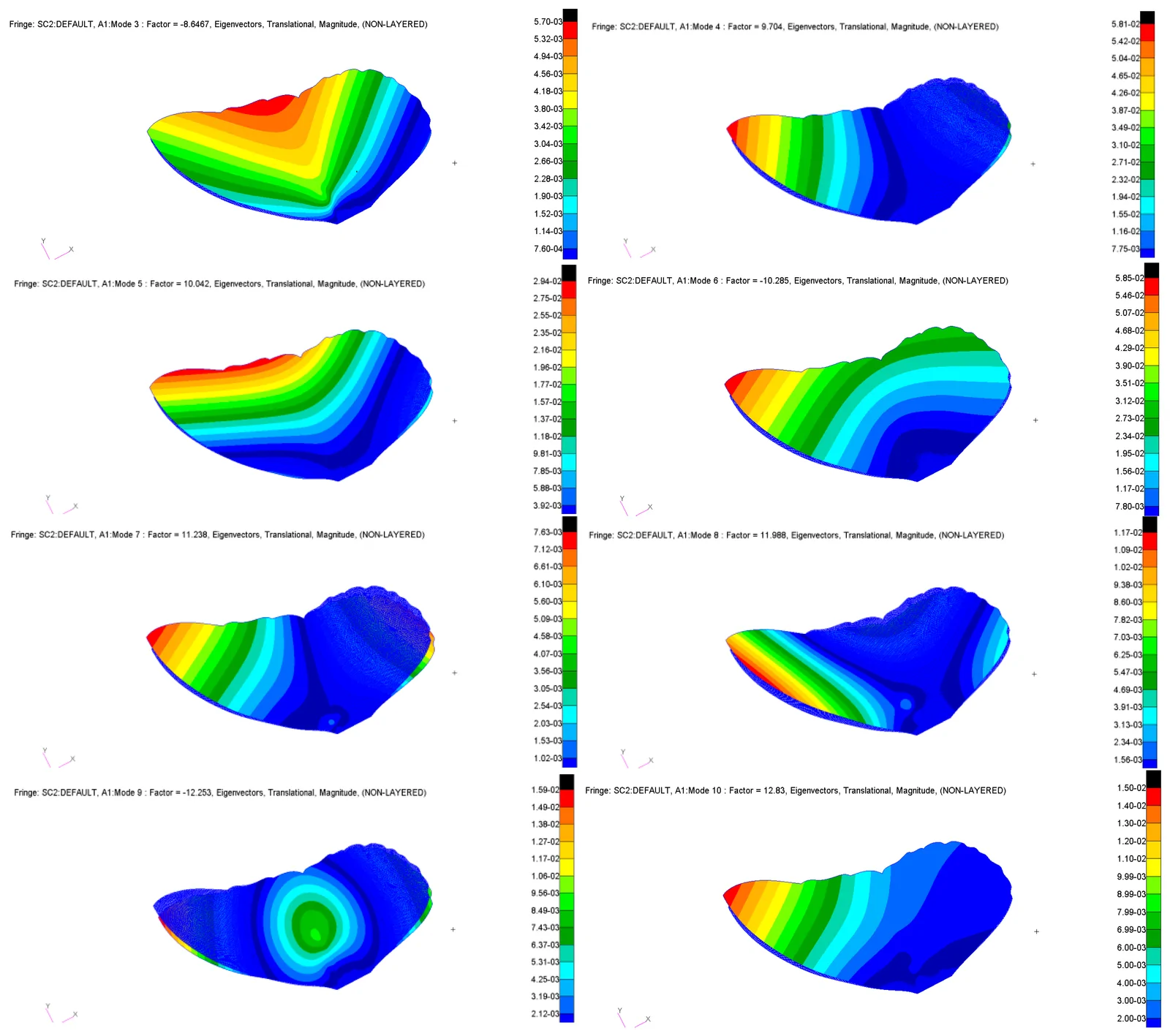
Buckling Modes of the First Ten Orders.

To investigate the stiffness performance of the butterfly wing membrane, the first ten orders of buckling mode analysis were carried out, as shown in Figure 11.

The description of the first-10 buckling modes is shown in Table 4.

According to Section 2.2, the critical buckling factor is 7.1642. It is greater than 1, indicating that the butterfly wing membrane is free of buckling failure. Insufficient stiffness only results in relatively large displacement and rotation angle at the wing tip.

#### 3.2.4. Structural Modal Optimization Analysis

Considering that butterflies need to increase wing membrane stiffness during evolution, we have fine-mesh model above.

Combes & Daniel [46] detached insect wings at the basal hinge and performed mechanical tests with minimal clamping to characterize the inherent bending stiffness of the wing membrane and vein network. This approach decouples the intrinsic mechanical properties of the wing itself from the elastic constraints introduced by the basal hinge.

**Figure 12:**
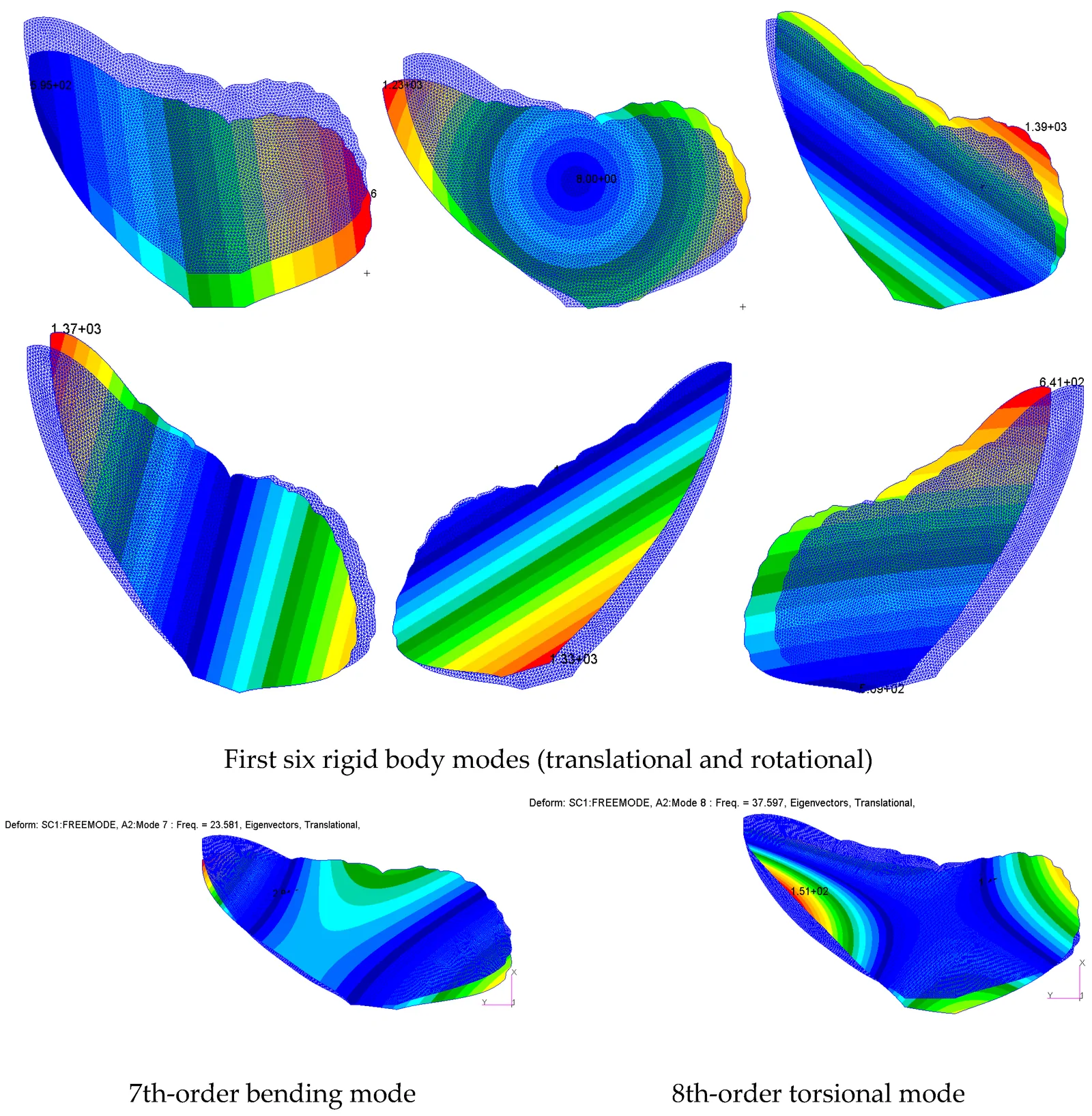
Mode Shapes of the Refined Finite Element Model.

Modal analysis was performed on the above model, and the first 8 mode shapes are shown in Figure 12 (first six rigid body modes—translational and rotational; 7th-order bending mode; 8th-order torsional mode).

Vibration-Mode Characteristics of the wing membrane is shown in Table 5.

Under free-free boundary conditions, an unconstrained solid structure yields six zero-frequency rigid-body modes, including three translational rigid-body modes and three rotational rigid-body modes. These modes represent overall kinematic motion of the whole model without internal elastic strain. Real elastic vibration modes begin at Mode 7.

**Figure 13:**
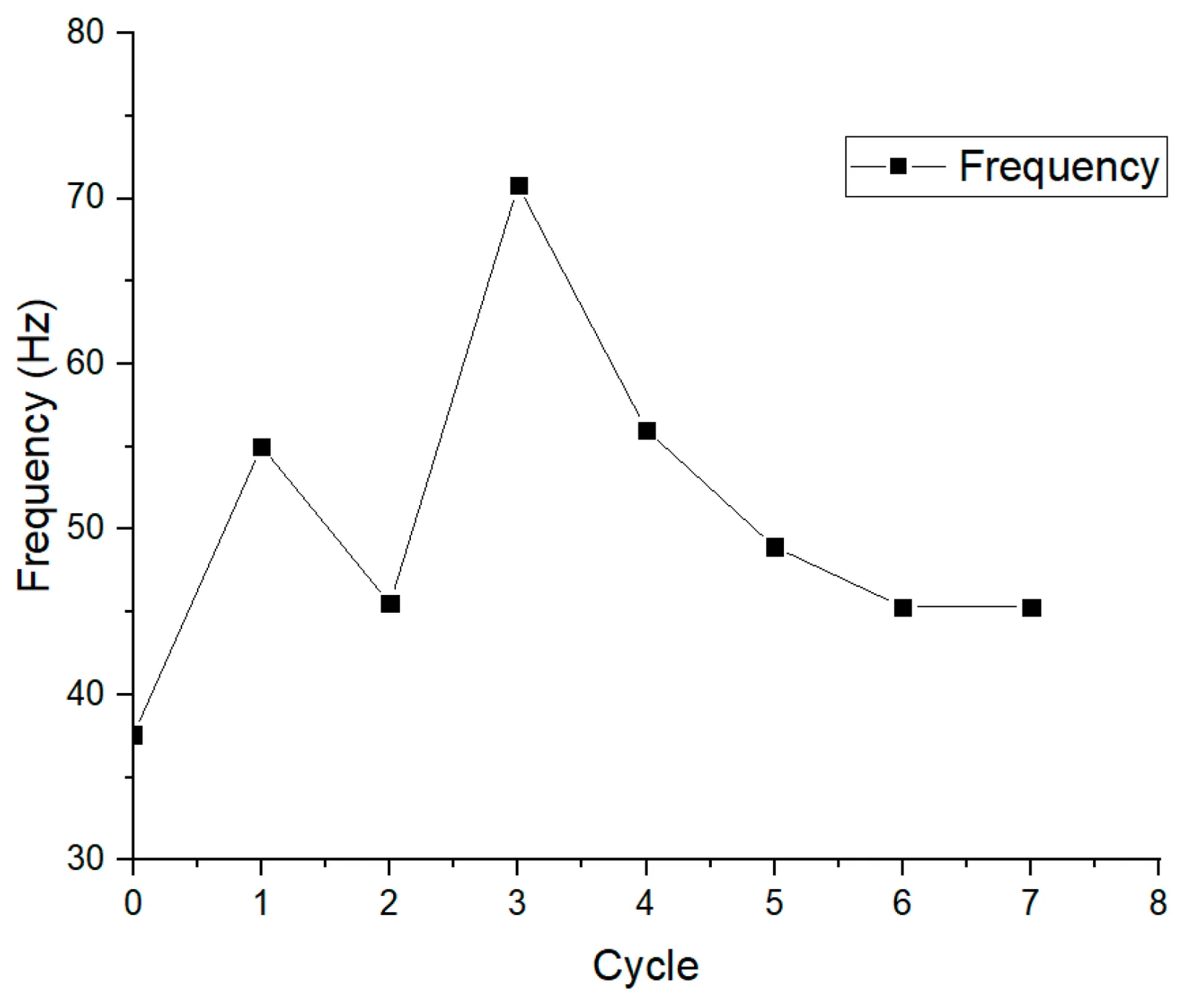
Optimization History.

After 7 iterations, run terminated due to hard convergence to an optimum at cycle 7 (see Figure 13). The frequency can be increased to approximately 45.3 Hz.

**Figure 14:**
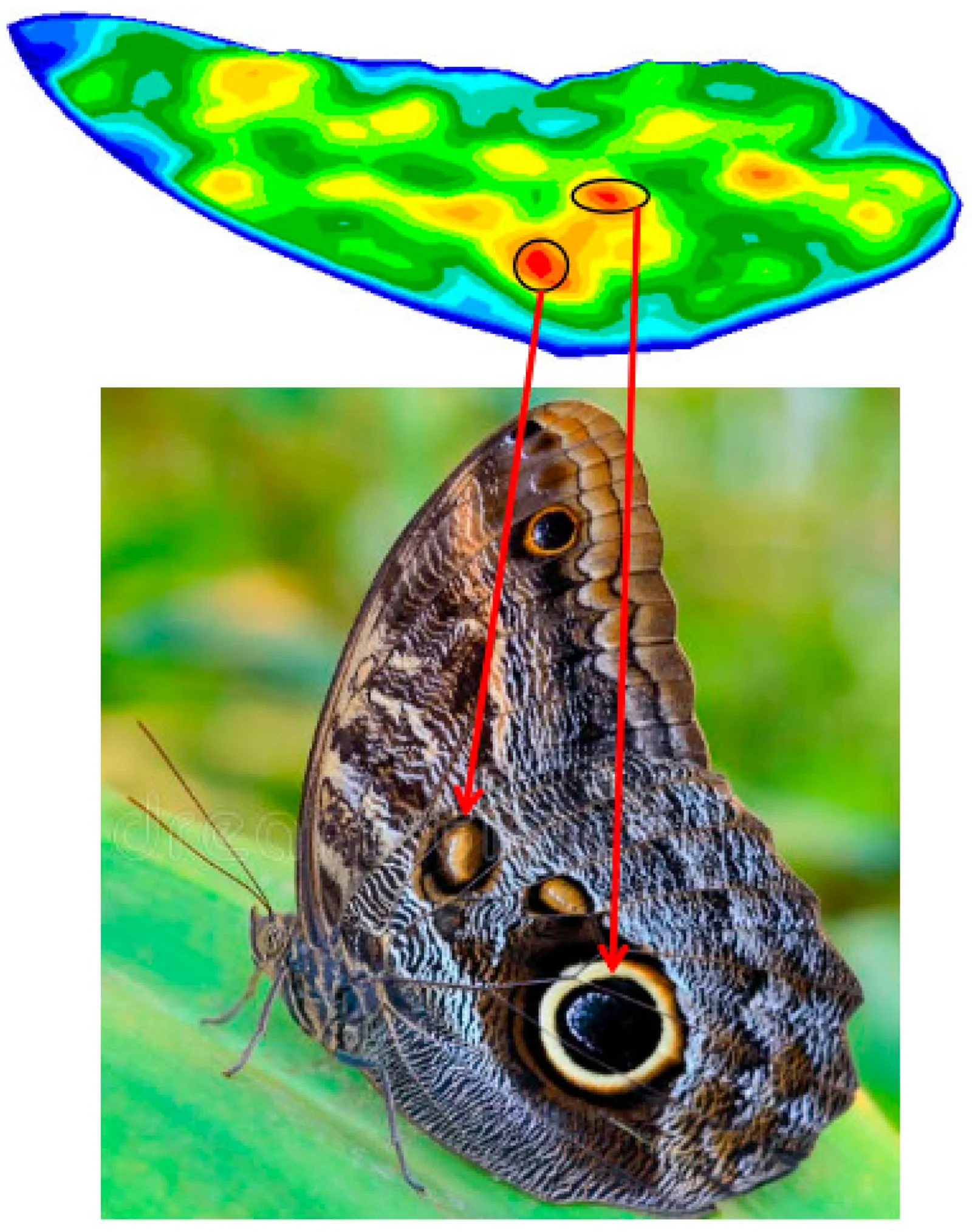
Morphology of the Wing Membrane for Enhanced Stiffness [48].

The shape of the wing membrane at cycle 7 can be seen in Figure 14.

The protruding part in the simulation diagram is somewhat similar to the thickened shape of the black spots on butterfly wings, indicating that the thickened structure of these black spots serves an additional function: it increases the stiffness of the butterfly wing membrane and raises its natural vibration frequency.

These numerical results are consistent with the biochemical, morphological and geometric mechanisms reviewed in Section 1.3.2. Melanin-rich black spots are more thoroughly sclerotized and thus mechanically stiffer [30,32,33], the scales in these regions possess more elaborate cuticular architecture [34], and the local geometric relief produced by the thickened spots raises bending resistance in a manner analogous to wing corrugation and venation [36,38,39]. The optimal profile predicted by topography optimization therefore converges on the same structural strategy that butterflies have evolved: concentrating material at the most deformable regions of the membrane to maximize stiffness with minimal mass increase. This agreement between an optimization-based prediction and natural morphology provides partial support for the stiffness-enhancing function of black spots proposed here.

### 3.3. Discussion

#### 3.3.1. Multi-Functional Integration of Black Spots

The present study adds a mechanical dimension to the well-established biological functions of butterfly black spots. While previous work has emphasized their roles in predator defense [27,28,29], thermoregulation [30,31] and signaling [22,23], the numerical results here, together with the biochemical [30,32,33], morphological [34,35] and geometric [36,37,38,39,40] evidence, indicate that black spots also act as localized stiffness enhancers that raise the natural frequency of the wing membrane. This multi-functionality—optical, thermal and mechanical roles integrated into a single structural feature—is a hallmark of biological “material-efficient” design and suggests that bionic wing membranes should deliberately incorporate such pigmented, thickened features rather than treating coloration and stiffness as independent design variables.

#### 3.3.2. Implications for Flapping-Wing Simulation and Design

Existing flapping-wing simulations typically idealize the membrane as a uniform, smooth flexible material [2,3,4], which neglects the stiffening contribution of local morphology. The present results demonstrate that such morphological details measurably affect the natural frequency and stiffness of the membrane and therefore should be incorporated into high-fidelity FSI models to improve simulation accuracy. The black-spot-inspired “local thickening” design derived from topography optimization provides a lightweight, manufacturable strategy for tailoring the stiffness distribution of biomimetic membranes and could serve as a practical guideline for the structural design of micro flapping-wing vehicles.

#### 3.3.3. Limitations and Future Work

Several limitations should be acknowledged. First, the present analysis is purely numerical and is based on idealized material parameters (Table 2) and a representative butterfly geometry; the stiffness-enhancing function of black spots has not been directly measured in real wings. Nevertheless, the effect is strongly supported by independent experimental evidence from cuticle sclerotization [32,33], scale morphology [34], wing corrugation [36] and venation [38], which provides credible indirect support. Direct flexural-stiffness mapping of natural wings (e.g., following the point-load method of Combes and Daniel [49]), Rigid-flexible Coupling Wing Design in the MAV by Li Chen-Chen et al. [50] and systematic comparisons between spotted and unspotted wing regions would provide further quantitative validation. Second, the large-scale material parameters in Table 2 were adopted for a preliminary exploration, and their influence on the optimized topography deserves a sensitivity study. Third, the topography optimization was performed on a static membrane under a representative pressure load; coupling with full flapping kinematics and unsteady aerodynamic loads is left for future work.

## 4. Conclusions

Bionics is an ancient yet young discipline with a time-honored theoretical origin. By imitating the structures and functions of organisms, it provides innovative principles, methods and research pathways for human engineering and technological systems. After the long evolutionary process of nature, butterflies have become an important research subject in bionics due to their unique flight patterns and gorgeous wing surface morphology. From aerodynamic load analysis to structural strength investigation, and from wing membrane topography optimization to the exploration of mechanical mechanisms underlying morphogenesis, every research dimension reveals the profound wisdom of natural creation to mankind.

As an emerging research field, topology optimization design shares a highly consistent core philosophy with the structural logic of butterfly wings: materials are only arranged at critical load-bearing positions to achieve optimal mechanical performance with the simplest configuration. Wing membrane topography optimization is a direct embodiment of this design concept. In terms of morphological characteristics, the research value of butterfly wing membranes lies not only in their macroscopic appearance but also in the sophisticated configuration of their surface topography. There are more than 14,000 species of butterflies worldwide with diverse wing shapes, which exhibit hierarchical structures featuring multi-morphology, multi-scale, multi-dimensional and interdisciplinary characteristics, offering an extensive natural template library for the research and development of biological materials.

Starting with the extraction of morphological features of butterfly wing membranes, this paper carries out flight load simulation, structural strength and stiffness analysis, and planar topography optimization of wing membranes. From the perspective of mechanical mechanisms, it reasonably elucidates an alternative formation mechanism for the black spot structures on butterfly wings: enhancement of the structural stiffness of butterfly wings. Localized topographical thickening can increase the stiffness or natural frequency of the modeled membrane, and this may be qualitatively consistent with certain morphological features observed in natural butterfly wings. This stiffness-enhancing hypothesis is also consistent with the biochemical, morphological and geometric mechanisms reported in the literature, including melanin-coupled cuticle sclerotization, scale microarchitecture and wing corrugation. The above mechanisms collectively support the multifunctional nature of such natural structures and provide a biological basis for the morphology–stiffness coupled design of biomimetic flapping-wing membranes.

## 5. Final Remarks

In future research, the enhancement of butterfly wing stiffness through venation arrangement will be taken as the research focus, and further multidisciplinary optimization will be carried out on the existing flapping-wing simulation model. This aims to control the errors between simulation results, real biological flight states and physical test data within an acceptable range, as well as improve the accuracy and reliability of flapping-wing simulation. Meanwhile, a bionic wing design method based on the morphological characteristics and mechanical mechanisms of biological wings will be established, providing theoretical and technical support for the structural optimization of wings of flapping-wing aircraft.

In future practical applications, this study can offer a theoretical basis and technical support for the research and development of products such as micro flapping-wing aircraft and special detection flapping-wing equipment. It will promote the transformation of flapping-wing bionic technology from laboratory research to engineering application, elevate the technical level in the field of flapping-wing aerospace bionics, and further expand the application scenarios of flapping-wing technology.

## Figures and Tables

**Table 1:** The material mechanical parameters of the butterfly structure [45,46,47].

Mechanical Parameter	Value Range	Unit
Longitudinal elastic modulus (E1)	4.0	GPa
Transverse elastic modulus (E2)	1.2	GPa
Longitudinal-transverse shear modulus (G12)	1.0	GPa
Out-of-plane Shear Modulus (G23, G13)	0.2	GPa
Tensile Strength (σt)	30–80	MPa
Elongation at Break (εb)	5–15%	–
Poisson’s Ratio (υ)	0.3–0.35	–
Density (ρ)	1.2–1.4	g/cm^3^

**Table 2:** Support reaction forces.

Load Type	Fx	Fy	Fz
Value (N)	−8.287505 × 10^−8^	3.669327 × 10^−2^	3.951820 × 10^−2^

**Table 3:** Comparison of numerical results with different mesh densities.

Number of Elements	Maximum Stress (Pa)	Maximum Wing-Tip Deformation (mm)	First Elastic Natural Frequency (Hz) *
1962	1.02 × 10^7^	7.24	20.58
7848	1.18 × 10^7^	7.28	22.0618
31,392	1.28 × 10^7^	7.3	23.581

* The mode shape of the first elastic natural frequency is shown in Figure 12.

**Table 4:** Naming and characteristics of the first-10 buckling modes.

Mode No.	Buckling Factor	Mode Shape Description	Mode Designation
Mode 1	7.1642	Normal bulging deformation occurs in the leading-edge region, while the trailing-edge remains nearly stationary. It is a one-order local bending buckling with unidirectional local protrusion.	**Mode 1: Local outward bulging buckling at leading-edge**
Mode 2	−7.663	The leading-edge caves inward, with smoothly continuous deformation distributed along the span-wise direction; displacement at the trailing-edge is negligible. The deformation direction is opposite to Mode 1.	**Mode 2: Local inward-concave buckling at leading-edge**
Mode 3	−8.6467	Large-scale inward depression appears in the middle-front region. The deformation center locates at the mid-front of the structure, forming a single-valley concave pattern in the span-wise direction.	**Mode 3: Global inward-concave buckling of mid-front section**
Mode 4	9.704	Local outward bulging recurs at the leading-edge. Compared with Mode 1, the bulging area expands slightly and the trailing-edge remains rigid.	**Mode 4: Extended-range outward bulging buckling at leading-edge**
Mode 5	10.042	Large-scale span-wise bending occurs, with smooth single-wave outward-protruding deformation transitioning from leading-edge to the middle area.	**Mode 5: First-order global outward-protruding bending buckling in span-wise direction**
Mode 6	−10.285	Large-scale span-wise inward depression. Its deformation shape is opposite to Mode 5, presenting a single-valley pattern covering the leading-edge to the middle region.	**Mode 6: First-order global inward-concave bending buckling in span-wise direction**
Mode 7	11.238	Local outward bulging at the leading-edge, accompanied by local warping near the structural root; two deformation-concentrated zones are observed.	**Mode 7: Composite buckling of leading-edge bulging plus root local warping**
Mode 8	11.988	Local bulging at the leading-edge together with secondary local protrusion near the root; buckling coupling occurs in two separate local regions.	**Mode 8: Dual-zone coupled local buckling (leading-edge-root)**
Mode 9	−12.253	Annular depression emerges at the structural center, and displacement decays radially outward. It corresponds to local pit-type instability in the central zone.	**Mode 9: Local pit-type inward-concave buckling at central region**
Mode 10	12.83	Outward bulging of the leading-edge with gradient span-wise deformation; the trailing-edge shows almost no displacement. Single-wave outward-protruding deformation mainly concentrates on the front half of the component.	**Mode 10: Span-wise single-wave outward-protruding buckling of front-half section**

**Table 5:** Descriptions of the first eight vibration modes for the wing membrane.

Mode No.	Mode Name	Mode Shape Description
Mode 1	Rigid-body translational-rotational mode 1	Rigid-body motion without structural deformation; the whole component undergoes pure rigid translation/rotation, no relative displacement between internal elements.
Mode 2	Rigid-body translational-rotational mode 2	Global rigid-body motion. Contour gradient represents pure rigid displacement; no local elastic bending or torsional deformation occurs on the curved surface.
Mode 3	Rigid-body translational-rotational mode 3	Entire structure moves as a rigid body. Displacement magnitude distributes continuously over the geometry, free of elastic strain inside the component.
Mode 4	Rigid-body translational-rotational mode 4	Pure rigid-body motion. The mesh maintains its original shape; colour fringe only reflects rigid-body offset rather than elastic deformation.
Mode 5	Rigid-body translational-rotational mode 5	Global rigid-body rotation or translation. No bending distortion is observed for the curved-surface geometry.
Mode 6	Rigid-body translational-rotational mode 6	Sixth rigid-body mode. The structure exhibits rigid-body kinematic behaviour with zero elastic deformation.
Mode 7	7th-order bending mode	Elastic bending deformation. Obvious span-wise flexural distortion appears on the curved surface; displacement concentrates in partial regions of the shell, representing the first elastic bending mode after eliminating rigid-body modes (frequency = 23.581 Hz).
Mode 8	8th-order torsional mode	Elastic torsional deformation. The curved-surface shell undergoes twisting distortion; two separate displacement-concentrated zones can be identified, corresponding to the fundamental elastic torsional mode (frequency = 37.597 Hz).

## Data Availability

The datasets generated or analyzed during the current study are available from the corresponding author on reasonable request. All simulation parameters and calculation procedures are clearly described in the manuscript to ensure reproducibility.
